# Nanotechnology and Pediatric Cancer: Prevention, Diagnosis and Treatment

**Published:** 2015-12-10

**Authors:** H Zare-Zardini, A Amiri, M Shanbedi, A Taheri-Kafrani, Z Sadri, F Ghanizadeh, H Neamatzadeh, R Sheikhpour, F Keyvani Boroujeni, R Masoumi Dehshiri, A Hashemi, MM Aminorroaya, MR Dehgahnzadeh, Sh Shahriari

**Affiliations:** 1 Department of Biotechnology, Faculty of Advanced Sciences and Technologies, University of Isfahan, Isfahan, Iran; 2 Hematology and Oncology Research Center, Shahid Sadoughi University of Medical Sciences and Health Services, Yazd, Iran; 3 Department of Mechanical Engineering, Faculty of Engineering, University of Malaya, Kuala Lumpur, Malaysia; 4 Department of Chemical Engineering, Faculty of Engineering, Ferdowsi University of Mashhad, Mashhad, Iran; 5 Department of Biology, University of Science and Arts, Yazd, Iran; 6 Shahid Sadoughi Hospital, Shahid Sadoughi University of Medical Sciences and Health Services, Yazd, Iran

**Keywords:** Cancer, Children, Functionalization, Nanotechnology

## Abstract

Despite development of new approaches for the treatment of cancer disease, it is the second cause of mortality in world. Annually, 30000 persons die in Iran due to cancer diseases. Eighty percent of cancer patients are children which about 50% children lead to death. Given the high rate of cancer-related death, the new approaches for prevention, control, early diagnosis, and treatment of this disease seem necessary. Investigation of new strategies is the major challenge for scientists at recent century. Nanotechnology as a new scientific field with novel and small compounds utilized different fields over the past ten years especially in medicine. This science has come to the forefront in the areas of medical diagnostics, imaging, and therapeutic scheduls. Therefore, it has the potential applications for cancer detection and therapy. This review will discuss the therapeutic applications of different nano-materials in diagnosis, imaging, and delivery of therapeutic agents for the treatment of cancer with a major focus on their applications for the treatment of cancer and cancer- related diseases in children. The advancements in established nanoparticle technologies such as liposomes, polymer micelles, and functionalization regarding tumor targeting and controlled release strategies as well as drug delivery were discussed. It will also review the blood toxicity of used nanostructures.

## Introduction

One of the most fatal diseases in human beings is cancer which annually leads to death of 30000 persons just in Iran. Eighty percent of cancer patients are children which about 50% children lead to death. Different researchers try to eradicate this deadly disease through different ways. So far, several compounds with anticancer effects are presented. The use of nanomaterials was also considered in this context. The use of nanotechnology can affect many aspects of human life (1). One of these aspects is medicine. Construction of nano compounds with different biological effects and their use in various industries are key field in science (2). The use of nanostructures in the cancer research has become active trend and leads to introduce a novel filed of nanotechnology: Cancer Nanotechnology (3-5). 

Cancer Nanotechnology research is interdisciplinary and incorporates biology, chemistry, engineering, medicine, and physics. Targeted delivery of chemotherapic drugs is the major reason for the development of cancer nanotechnology (2, 3). The research showed that 99% of chemotherapy drugs do not reach to the cancer cells. The release of these toxic drugs can damage normal cells (6, 7). All of chemothrapic drugs have different adverse side effects such as fatigue, pain (headaches, muscle pain, Stomach pain, and pain from nerve damage), mouth and throat sores, diarrhea, nausea and vomiting, constipation and blood disorders (8-11). Nanostructures including nanotubes, nanorods, dendrimers, nanospheres, nanotubes, coantum dots and etc can be used for passive and active targeting (12-15). This targeting leads to reduction of side effects of chemotherapic drugs. Nanostructures can be used in all cancer stages research: prevention, diagnosis and treatment (16, 17). This paper will review the therapeutic applications of nanotechnology in all of these stages, specially treatment of cancer disease with a major focus on their applications for the treatment of pediatric cancer. The blood toxicity of applied nanostrcutures is also discussed. 

**Nanomedicine**

One aspect of the application of nanotechnology is in the medical field. Nanotechnology is used in effective drug delivery systems, tissue engineering, cancer therapy, gene delivery, diagnostic techniques, antimicrobial techniques, cell repair and etc (18-24). Nanotechnology can deliver medicine or drugs into specific parts of the human body and thus increase effectiveness and reduce harmfulness. Nanoparticles, carbon nanostructures, liposomes, micelles and other nanostructures are useful to treatment of diseases. Surgical tools and robots can be made and used with nanotechnology to perform microsurgeries on any part of the body. This surgery doesn’t damage a large amount of the body. These microsurgerical tools would be precise and accurate, targeting only the area where surgery should be done. Nanotechnology can also used for the improvement of surgery visualization. Nano-robotics is other field of nanotechnology that use in medical fields including diagnosis and targeted drug delivery, surgery, pharmacokinetics, monitoring of diabetes, and health care. The important use of nanomaterials is as drug and drug delivery system in cancer (25-27). Nanotechnology was used in prevention, diagnosis and treatment of cancer. However, cancer therapy by nanotechnology has mostly focused on targeting tumor cell, namely treatment of cancer (28-30). Nanotechnology Programs for cancer therapy was designed by National Cancer Institute (NCI) (http://nano.cancer.gov/) in the U.S. as Alliance for Nanotechnology in Cancer from 2004 including the current status of development, opportunities for growth, and clinical applications for the nanotechnology. 

**Nanotechnology in Cancer Prevention**

The first strategy in cancer research is prevention (31). The main cause of cancer is carcinogenic substances as well as oxidant agents. Carcinogens affect DNA and lead to mutation (32, 33). So, the first strategy for cancer prevention is removing of carcinogenic substances. We can’t prevent this problem by the present technology. The elimination of carcinogens is an effective way for cancer prevention. The nonobiotechnology, among different sciences, can be use in cancer prevention. However, there is little research on preventive treatments using nanotechnology. Nanostructures can eliminate the carcinogen and oxidant agents from human environmants. The one possible preventive strategy is attachment of nanoparticles to UV scattering substances like zinc oxide (ZnO) and titanium oxide (TiO2), or UV absorbing substances like octyl methoxycinnamate and oxybenzone, and specifically target these nanoparticles to skin cell surface proteins and coating cells by these engineered nanostructures. By this nanotechnology-based preventive treatment method, it’s possible to eliminate most of the problems that occure by Carcinogens (34). 

**Nanotechnology in Cancer Detection**

Rapid and effective diagnosis of cancer lesions are one of the goals of medical science. Since current technologies in cancer imaging cannot provide appropriate differentiation of first detection based on lesion, nanotechnology can be considered as a good candidate for detecting cancer in early stages (35-37). This success is achieved through evolution of fluorescent probes named as Quantum Dots (QDs). Modified QD can be used as strong immunological probes to detect cancer markers such as Her2 and as other cell targets in putative tumor cells (38). Due to stability of the particles, long-term records of investigations on cells for fluorescent information are possible (39). Nanowires can be used as extremely small biological and chemical sensors (40, 41). Other achievements of researchers in this field are tool manufacturing for diagnosis of antigen specific for prostate cancer, PSA-α1antichymorypsin, carcinoembryonic antigen, and mucine in serum samples (42). Another application in detecting the presence of telomerase is siliceous nanowires. For this purpose, the siliceous nanowires were bound to telomerase using oligonucleotide supplement, and at the time of diagnosis, the cells, which have telomerase ,connect to them and change the conductivity of the nanowires, so that the presence of telomerase is recognized (43-45). The other nano devices in determining biomarkers are carbon nanotubes (CNTs) (46). Specific sequences of DNA can be detected from sequences with a single mistake using single-wall carbon Nanotubes (SWCNTs) as tip of Atomic Force Microscope (AFM) (47). The researchers synthesized nanoparticles that are able to detect blood clots and cancer cells. They cover nanoparticles surface with peptides. These peptides were digested by protease produced by cancer cells. Injected into the body, the particles are deliverd to tumors through blood vessels. When reached to the tumors, particles are digested quickly by proteases. These small particles entered into the blood stream and eventually excreted from the body through urine. Urine samples are tested for the detection of particles and peptide' components. The cancer is identified through the amount and type of peptides in the urine. Nanosensors are very delicate and sensitive tools that able to identify and respond to physical stimuli. There are several nanobiosensoers such as nanowires, nanocantilevers and quantum dots were developed (48). The most famous example of nanosensors that use in medicine is cadmium selenide (CdSe). This nanosensor can detect cancerous tumors using fluorescence characteristics.

**Nanotechnology in Cancer Treatment**

Since the existing chemotherapy treatments do not distinguish between cancerous and healthy tissue, designing nanoparticles that can distinguish such difference is important. Nano-materials are recently used to bind specific ligands to cancer cells. Due to the increasing rate of drug resistance in cancer patients, the use of nanoparticles as drug carriers to improve anticancer drug delivery is recently proposed. Nanotechnology can target delivery of drugs, genes, and proteins in the tumor tissue; therefore, it reduces toxicity of anti-cancer agents for normal tissues (49-51). Inactivation of drugs in biological environments would be prevented by importing pharmaceutical components into the nano-capsule. Lectins, ligands, and cell-specific antibodies can also be used to target tumor cells (52, 53). Using metal nanoparticles such as zinc oxide in the treatment of cancer optical dynamics is another treatment based on nanoparticles (54-57).

**Types of Nanostructures in Cancer Therapy**

**Liposomes**

Liposomes can use as delivery vehicle for delivering agents to target cells (58-60). Up to the present time different liposome-based drugs were developed and approved for cancer therapy (61, 62). Using liposome in drug delivery raises specificity of drug actions on tissues; in addition it reduces side effects of drugs on other tissues resulted in greater safety and specificity of drugs (63). The most important of these liposomal systems were summarized in Table I. All of these delivery systems have exhausted the clinical trials stages and some of them were approved by FDA.

**Micelles**

Drug delivery based on micelles was acquired by two different methods: passive and active drug deliverys (87, 88). Two important applications of micelles are delivery of contrast agents to target cells for imaging and drug delivery, especially in cancer therapy (89, 90). Now, polymeric micelles loaded with anticancer drugs are being investigated in pre-clinical studies for treatment of cancer and enhancement of drug effect (91, 92). The results show that polymeric micelles are also good candidate for gene delivery (93-95). The most important of polymeric micelles that are used for drug delivery and are being investigated in pre-clinical studies are summarized in Table II.

**Magnetic Nanoparticles**

Magnetic nanoparticles gain high practical importance in the diagnosis and treatment of cancer because of possessing special magnetic properties. Through investigating sources and multiple studies conducted in this field, it can be concluded that the load of anticancer drugs on the magnetic nanoparticles plays an important role in enhancing drug performance and eliminating the cancer cells. The results showed that the magnetic nanoparticles reinforce the performance of anticancer drugs such as doxorubicin to kill cancer cells by strengthening the production of reactive oxygen species or other unknown mechanisms (100). In other words, the magnetic nanoparticles enhance the cytotoxic anticancer drug, and also play an important role in drug delivery to tumor cells (101, 102). Among magnetic materials, nanoparticles of iron oxide are only the magnetic materials which pose suitable characteristics for being used in the medical environment. The articles showed that iron oxide nanoparticles have no immediate or long-term in vivo toxic effect (103, 104). These magnetic nanoparticles are very important for medical diagnosis applications such as increasing the contrast of magnetic resonance imaging (105, 106). 

**Carbon Nanotubes**

So far, various research groups applied carbon nanotubes for the development of targeted anticancer drugs. The scientists have shown that carbon nanotubes can transfer proteins and anti-cancer drugs into cells. CNTs can be used as a therapeutic agent. For example, carbon nanotubes can act as nano-bombs and lead to disintegration of cancer cells. It is possible to use nano-bombs carbon as effective therapeutic factors for killing cancer cells. According to different articles, the waves emitted from the explosion of these bombs not only eradicate cancer cells, but also all of the small vessels feeding. As soon as the bomb exploded in situ and cancer cells were destroyed, immune cells (macrophages) effectively digest and eliminate cellular debris and exploded nanotubes with circulatory system (107, 108). CNTs were also used for detection of tumor markers such as PSA, CEA, AFP and etc (109, 110). 

**Quantum Dots**

Bioconjugated Quantum Dots (QDs) fluorescent probes are able to present a promising and powerful image for cancer detection and diagnosis (111, 112). Many new techniques have been immerged during the past decade in order to apply the unique photophysical properties of QDs, for the in vitro biomolecular profiling of cancer biomarkers, in vivo tumor imaging, and dual – functionality tumor - targeted imaging and drug delivery. Currently some of these emerging technologies are being improved and integrated into clinical practice in oncology which can lead to important implications for the diagnosis, prognosis, and therapeutic management of cancer patients in the near future (38, 113-115).

**Dendrimers**

Dendrimers can be applied to a variety of cancer therapies to improve their safety and efficacy. These nanostructures also use in photodynamic therapy and gene therapy for cancer treatment (116, 117). These fundamental advances can present highly versatile and potentially powerful technologies for drug delivery when coupled with practical methods to covalently conjugate a wide range of bioactive molecules to the surface of a dendrimer or encapsulate them as guest molecules within void spaces (118). Dendrimers can also use as contrast agents for cancer diagnosis by imaging techniques such as MRI (119). 

**Nanotechnology and Pediatric Oncology**

**Nanotherapy of Pediatric Leukemias**

Two major forms of blood cancer are acute lymphoblastic leukemia (ALL) and acute myeloid leukemia (AML). Despite the marked successes in ALL and AML treatment, the prognosis and treatment of patients with ALL and AML is big challenge regarding of target toxicity and residual effects of chemotherapy (120, 121). Recently, application of nanostructures such as dendrimers, gold particles, liposomes, micelles, and polymers has been investigated and described (122). These agents can improve the availability and therapeutic efficacy of conventional drugs (123, 124). The one example of this delivery system is Marqibo. Marqibo is a liposomal formulation of vincristine that approved by FDA in 2012 for certain Philadelphia chromosome-negative (Ph)-ALL patients (125, 126). 

**Nanotherapy of Pediatric Bone Cancer**

Osteosarcoma is the most important bone cancer that occurs in children and usually affects the large bones of the arm or leg (127). Chemotherapic agents and surgery have been improved the treatment of patients with osteosarcoma so that the 5-year survival rate was increased from 15% to 65% (128). But, the metastatic diseases remain the most challenging and effects on overall survival rate (129). Nanotechnology has the highest opportunity to deliver high doses of cytotoxic agents to osteosarcoma cells and decrease side effects on normal tissues. 

**Nanotechnology and Immune Deficiencies Treatment**

Chemotherapy drugs cause immune deficiency following cancer treatment that lead to development of infectious diseases, especially in children. Indiscriminate use of antibiotics for treatment of these infections leads to development of resistant microbial strains (130, 131). This status is more than important for children’s infections. Nanostructures are suitable candidates for prevention of infections in children with immunodeficiency (132). The best strategy for prevention of infection is coating of surfaces by antimicrobial agents, especially nanostructures. Nanomodified surfaces reduce bacterial growth. Different nanostructures can be coated on the surface of medical devices, window, wall etc., especially for isolation room in pediatric section (133, 134).

**Toxicity of Nanostructures**

Recently, there is a rise in the number of publications about toxicity of nanostructures. Human tissues such as skin, lungs, and the gastro-intestinal tract are mostly exposed to environmental agents (135). While the skin is generally an effective barrier to foreign substances, the lungs and gastro-intestinal tract are more vulnerable (136, 137). The toxicity of nanostructures depends on size, aggregation, composition, crystallinity, surface functionalization and etc. Nanostructures enter the circulatory system and lead to arteriosclerosis, blood clots, arrhythmia, heart diseases, and ultimately cardiac death (138, 139). The most application of nanotechnology in pediatric cancer is use of nanostructures as delivery system in injection method. So, a further concern is related to these particles that use in injection systems. Thus, the blood structures, clearance of nanostructures and vessel coat can be important factors in the biodistribution of nanostructures (140-142). 

**Toxicity for Normal Tissues**

The penetration of large and small molecules to healthy tissue was done through small holes in the epithelial cells of vascular tissue with a size of about 45- 250 Å. Some tissues especially liver and spleen have larger pore size and more phagocytic cells. They lead to accumulation of nanoparticles in these tissues. So, nanoparticles exerted more toxicity on these tissues. 

**Blood Toxicity**

Different studies have shown that nanostructures cause hemolysis and blood clotting (143). Nanoparticle uptake by red blood cells and platelets is related to size and nanoparticle charge respectively (144). Thrombosis occurs during the first hour after exposure to different nanostructures (145). Cationic particles have more toxicity in comparison with anionic particles. The researchers indicated that nanoparticles can gain access to the blood following inhalation or instillation and then, they can enhance thrombosis, inflammation, and particles translocated to the blood (146). Campen et al showed that exposures to nanoparticles through inhalation can altered heart rate in hypertensive rats (147). Nanostructures inhibit thrombi formation and enhance platelet aggregation and thrombosis. Nanostructures interact with platelets and reduce their surface charge and lead to aggregation (148, 149). Zare Zardini et al showed that MWCNTs have lower blood toxicity than Ag nanoparticles.

These researchers and other scientists also revealed that functionalization of MWCNTs reduce toxicity on blood cells (150). According to studies, the functionalization of nanostructures by different compounds, especially biological agents, can enhance biocompatibilty of these nanostructures, in particular, blood compatibility (151-153). 

**Conclusion**

Prevention, diagnosis, and treatment of cancer are one of the major scientific challenges. This incurable disease continues to be a big problem in recent century. In recent years, many drugs and techniques have been developed for prevention, diagnosis and treatment of cancer including chemotherapy, radiotherapy, surgical removal, hyperthermia, and etc. Recently, it’s recognized that nanotechnology has potent properties for management of some problems in cancer therapy. Nanotechnology can be used in prevention, diagnosis, and treatment of cancer especially for early detection and effective drug delivery. Besides all the positive properties, nanostructures cause cytotoxicity and blood toxicity on normal cells and tissues such as thrombosis, inflammation, and etc. Different approaches especially functionalization provide the possibility for decreasing toxicity and enhancing properties of nanostructures. According to the findings of articles reviewed in this paper, nanotechnology and engineered nanostructures are suggested as effective cures for cancer in the near future.

**Table I:** Liposome-based drug delivery systems for cancer therapy

**Liposomal system**	**Application type**	**Refs**
**Liposomal doxorubicin **	Recurrent Ovarian Cancer, Metastatic Breast Cancer and Multiple Myeloma	(64-67)
**Non-pegylated liposomal doxorubicin **	Metastatic Breast Cancer	(68, 69)
**Thermally sensitive liposomal doxorubicin **	Hepatocellular Carcinoma	(70-72)
	Breast Cancer	
	Hepatocellular Carcinoma	
**Liposomal paclitaxel **	Solid tumours	(73-75)
	Breast Cancer	
	Pancreatic Cancer	
	Liver Cancer	
**Liposomal vincristine **	ALL	(76-78)
**Liposomal cisplatin **	Advanced or refractory solid tumours Pancreatic cancer, non-small-cell lung cancer, head and neck cancer and breast cancers Ovarian cancer	(79-83)
**Liposomal irinotecan **	Colorectal carcinoma Colorectal cancer Metastatic pancreatic cancer	(70, 84, 85)
**Liposomal vinorelbine **	Hodgkin's disease, non-Hodgkin's lymphoma	(86)

These date have aquierd from http://www.nano.ir

**Table III:** Developed polymeric micelles for chemotropic drug delivery in pre-clinical stage (http://www.nano.ir/)

**Micelles**	**Block**	**Used drug**	**Refs**
**NK012**	PEG-Pglu (SN38)	SN-38	(96)
**NK105**	PEG-P (Asparatate)	Paclitaxel	(97)
**SP1049C**	F127,L61, Pluronic L61and F127	Doxorobicin	(87, 88)
**NC6004**	PEG-Pglu (cisplatin)	Cisplatin	(98, 99)
